# Efficient Process Development of Recombinant Human Granulocyte Colony-Stimulating Factor (rh-GCSF) Production in *Escherichia coli*

**DOI:** 10.6091/ibj.1338.2015

**Published:** 2015-04

**Authors:** Valiollah Babaeipour, Sirwan Khanchezar, Mohammad Reza Mofid, Mahdi Pesaran Hagi Abbas

**Affiliations:** 1 *Dept. of Bioscience and Biotechnology, Malek Ashtar University of Technology, P.O. Box 14395-1561, Tehran, Iran; *; 2 *Dept. of Biotechnology, Chemical Engineering Faculty, Tarbiat Modarres University, Tehran, Iran;*; 3 *Dept. of Biochemistry and Bioinformatics Research Center, School of Pharmacy and Pharmaceutical Science, Isfahan University of Medical Sciences, Isfahan, Iran;*; 4 *Dept. of Life Science Engineering, Faculty of New Technologies, University of Tehran, Tehran, Iran*

**Keywords:** *Escherichia coli*, Granulocyte colony-stimulating factor (GCSF), Process development

## Abstract

**Background:**

The protein hormone granulocyte colony-stimulating factor (GCSF) stimulates the production of white blood cells and plays an important role in medical treatment of cancer patients.

**Methods:**

An efficient process was developed for heterologous expression of the human GCSF in *E. coli* BL21 (DE3). The feeding rate was adjusted to achieve the maximum attainable specific growth rate under critical value. In this method, specific growth rate was maintained at the maximum value of 0.55 h^-1^ at the beginning of feeding to 0.4 h^-1^ at the induction time. Recombinant human GCSF (rh-GCSF) was produced as inclusion body. At first, inclusion bodies were released by cell disruption and then washed, solubilized and refolded. Finally, the rh-GCSF was purified by cation exchange chromatography.

**Results:**

Obviouly, higher specific growth rate decreases process time and consequently increases productivity. The final concentration of biomass and GCSF was achieved 126 g DCW.l^-1^ and 32.1 g.l^-1^. Also, the final specific yield (Y_P/X_) and total productivity of rh-GCSF were obtained 254 mg.g^-1^ DCW and 1.83 g.l^-1^.h^-1^, respectively. According to the available data, this is one of the highest Y_P/X _and productivity that has been reported for any human protein which is expressed in *E. coli*. Recovery yield of purification process was %40 and purity of recombinant protein was over than 99%. The circular dichroism spectra of purified rh-GCSF, Neupogen® and PD-Grastim showed that all proteins have a similar secondary structure.

**Conclusion:**

Modified exponential feeding strategy for fed-batch cultivation of recombinant *E. coli, *results in minimum fed-batch duration and maximum productivity.

## INTRODUCTION

Human granulocyte colony-stimulating factor (GCSF) is a single chain polypeptide composed of 174 residues, (MW = 18.8 kDa). GCSF is a hemopoietic growth factor that stimulates proliferation, differentiation, and functional activation of blood cells. Active GCSF contains a free cysteine at position 17 and two intramolecular disulfide bonds formed between positions 36/42 and 64/74 [1-3]. 

It is well known that* E. coli* is a useful host for production of recombinant proteins [4-9], since it has simple nutrient requirement, well known molecular genetics and cellular physiology, and high growth rate on inexpensive substrate [4, 5, 9]. In the case of intracellular recombinant protein expression, productivity is proportional with the final cell density and the specific yield (i.e. the amount of product formed per time unit). Therefore, fed-batch cultivation, which is an important method to achieve high cell-density culture, improves productivity of target products. Suitable feeding strategies in fed-batch cultivation regulate available nutrient concentration and consequently affect specific growth rate, maximum cell concentration, the specific yield of recombinant protein, and formation of by-products [6, 10-12]. 

Exponential feeding is one of the most widely used approaches that allow implementation of the process and manipulation of specific growth rate [6, 12-14] by control of limiting substrate. In the exponential feeding method, cells grow under constant specific growth rate by increasing feeding rate corresponding to cell concentration and inhibit acetate formation by maintaining specific growth rate below the critical value. Therefore, keeping *μ* at a maximum attainable level and its control under a critical value before induction provide the nutrients at suitable concentr-ation ranges, which is very important for achieving high cell density and productivity [15,16].

Overexpression of heterologous proteins in *E. coli* cytoplasm often results in the formation of insoluble aggregates, so called inclusion bodies. Production of recombinant protein as inclusion body has a drawback and some benefits. Inclusion body contains high degree purity of target protein, and inactive proteins can be protected from proteolysis [17]. Also, it can be recovered from cell lysate (mechanically disrupted cell) by simple centrifugation [18]. The main challenge in recombinant protein production as inclusion body is the isolation of active proteins from inclusion bodies. This process includes inclusion body solubilization in high concentration denaturant agents (such as urea or guanidine hydrochloride) and protein refolding for remove of denaturant in the presence of oxidizing agent [19-21]. Generally, inclusion bodies are solubilized at extreme pH that is special for each protein. In the case of recombinant protein contain disulfide bond, appropriate redox condition is needed for protein refolding [17]. 

A few researches have shown that the recombinant human GCSF (rh-GCSF) expression level in *E. coli *is at moderate to high level [20,13], but the yield of final product is poor. Hence, for the first time in this study, we tried to obtain higher yield and productivity by keeping specific growth rate at a maximum attainable level during the exponential feeding of fed-batch cultivation. We also investigated the effect of feeding strategy on by-products and medium ingredient concentration, plasmid stability, and total process time in the fed-batch process during high cell-density culture of recombinant *E. coli * BL21 (DE3) [pET23a-hgcsf]. Then we tried to develop an efficient process for purification of rh-GCSF. Purified rh-GCSF was characterized with circular dichroism and size exclusion chromatography rather than standard samples, such as Neupogen® (Roche, Germany) and PD-Grastim (Pooyesh Darou, Iran). 

## MATERIALS AND METHODS

***Microorganism and vector system. ***The host cell, *E. coli* BL21 (DE3) (Novagen, United States), which contained pET23a inducible expression vector (Novagen, United States) carring the *rh-GCSF* gene [21] at *Not*I and *Nde*I sites. Transformed cells using the calcium chloride procedure were cultured on Luria-Bertani (LB) agar plates containing 100 mg/l ampicillin.

***Media and solutions. ***Transformed *E. coli *was cultivated on LB agar medium and M9 modified medium, which were used as seed culture and fermentation medium. M9 modified medium contained 10 g glucose, 15 g K_2_HPO_4_, 7.5 g KH_2_PO_4_, 2 g citric acid, 2.5 g (NH_4_)_2_SO_4_, 2 g MgSO_4_.7H_2_O, and 1 ml trace element solution per liter. The trace element solution consisted of 2.8 g FeSO_4_.7H_2_O, 2 g MnCl_2_.4H_2_O, 2.8 g CoSO_4_.7H_2_O, 1.5 g CaCl_2_.2H_2_O, 0.2 g CuCl_2_.2H_2_O and 0.3 g ZnSO_4_.7H_2_O per liter in 1 M HCl, and each medium contained 100 mg/l ampicillin. Fed-batch cultivation was carried out in a 2 L bench-top bioreactor (Inforse AG, Switzerland) with the working volume of 1 L, including two six-blade Rushton impellers with a speed range of 50-1,200 rpm.

***Analytical procedure. ***Cell growth was monitored by measuring culture turbidity at 600 nm and dry cell weight (DCW). For determination of DCW, pellet from 5 ml broth culture was dried at 105°C until constant weight [22]. Glucose, ammonia, phosphate, and acetate were analyzed enzymatically by using the appropriate kits (Chemenzyme, Iran and Boehringer Mannheim/R-Biopharm, Germany). The expression of rh-GCSF was analyzed by Coomassie brilliant blue-stained SDS-PAGE with 12.5% polyacrylamide [23] and quantified by a gel densitometer. Total soluble protein was analyzed by Bradford's method [24]. After SDS-PAGE, the gel was transferred to and blotted on the PVDF membrane in order to recognize rh-GCSF [23, 25]. The plasmid stability was determined by plating samples from fermentation broth on LB agar plates with and without ampicillin. Then the fraction of plasmid-containing cells on LB with ampicillin to those on LB without the antibiotic were calculated as plasmid stability [22]. 

***Fed-batch cultivation*** ***. ***A batch culture was initially established by the addition of 100 ml of an overnight-incubated seed culture grown at 32^o^C and 100 rpm (OD_600_ = 1.4-1.6). pH was maintained at seven by the addition of 25% (w/v) NH_4_OH or 3M H_3_PO_4_ solution. Dissolved oxygen was controlled at 30-40% (v/v) of air saturation by controlling both inlet air (which was enriched with pure oxygen) and agitation rate. Foam was controlled by the addition of silicon-anti-foaming reagent. After depletion of initial glucose in the medium, as indicated by a rapid increase in the dissolved oxygen concentration, the feeding was initiated. Feeding rate was increased step by step based on the exponential feeding strategy with maximum attainable specific growth rate during fed-batch cultivation. The exponential feeding was determined by the following equation (equation 1)    [26] :

(1) $$M\left(t\right)==F\left(t\right)S_{0}=[m+µ\frac{t}{Y_{\frac{x}{s}}}]S_{0}V_{0}X_{0}\exp[\int_{t_{0}}^{t}µ\left(t\right)\mathrm{dt}]$$

Where V_0_ is volume of the medium in the bioreactor (l), X_0_ is biomass concentration at the start of feeding g(DCW) l^-1^, t is the time (h), μ is the specific growth rate (h^-1^), S_0_ is the glucose concentration (g l^-1^) which is 400 g l^-1^ in the feeding solution, F(t) is feeding rate (l h^-1^), M(t) is mass feeding rate (g h^-1^), Y_X/S_ is the yield of biomass as a result of substrate (g DCW g^-1^ glucose), t_0_ (h) is the starting time for each feeding step, and m is the specific maintenance coefficient (g g^-1^ h^-1^). Feeding rate was corrected by the turbidity of taken samples every 10 minutes. 

The coefficient yield (Y_X/S_) and maintenance coefficient (m) were set at 0.5 and 0.025 g g^-1^ h^-1^, respectively. For development of a simple feeding strategy with the highest attainable specific growth rate during the entire process, a maximum oxygen transfer capacity was applied to the bioreactor, and glucose concentration was maintained below 2 g l^-1^ by a gradual increase in feeding at each step. 

Cells were induced by the addition of 2 mM IPTG in all experiments. The required nitrogen source (ammonium) was supplied by the addition of 25% (w/v) NH_4_OH, which was also used for maintaining pH at 7. The temperature of the process was maintained at 37ºC, and the acetate and glucose concentrations were controlled manually at 10-min intervals.

***Purification of*** ***recombinant human*** ***-granulocyte colony-stimulating factor. ***The fermented broth was centrifuged at 8,000 ×g at 4°C for 30 min, and the obtained pellet was washed twice with 50 mM phosphate buffer (pH 7.4). The wet cells (50 g per 200 ml lysis buffer) were suspended in lysis buffer, containing 100 mM Tris-HCl, 1 mM EDTA and 1 mM phenylmethylsulfonyl fluoride. Afterward, the cells were broken by passing the suspension through a homogenizer three times at 800 bar (Niro Soavi, North America). The cell homogenate was centrifuged, supernatant was discarded, and inclusion bodies were recovered. 

Next, inclusion body concentration was measured using Bradford's method [24]. Then it (1 g inclusion body per 5 ml) was resuspended in first washing buffer (2.5 g l^-1^ Triton X-100, 50 mM Tris-HCl, 5 mM EDTA, 1 mM phenylmethylsulfonyl fluoride [pH 8], and 0.01 μg/ml DNase1) incubated for 40 min and recovered by centrifugation at 8,000 ×g at 25°C for 30 min. Second washing step, the inclusion body pellet, was resuspended in second washing buffer (2 M urea) and incubated for 40 min and then recovered at 8,000 ×g at 25°C for 30 min. Then, 350 mg of washed inclusion bodies was dissolved in 10 ml inclusion body solubilization buffer (30 mM Tris-HCl [pH 12], 1 mM EDTA, 8 M urea, and 100 mM reduced glutathione. The solution was incubated at 25-28^o^C for 45 min and spun down to remove insoluble cell debris. The rh-GCSF was refolded by adding protein solution to refolding buffer (3 mg protein per ml refolding buffer with stirring), containing 30 mM Tris-HCl (pH 5), 3 M urea, 20 mM glutathione, 2 mM oxidized glutathione, and 1 mM EDTA). The pH of refolded protein solution was adjusted to 5 with 2 M acetic acid. Subsequently, refolded protein solution was applied in cation exchanger, Tricorn mono S 10/100 GL column (GE, USA), which was previously equilibrated with three bed volume of buffer A (20 mM Na-acetate, pH 5.0) at 4 ml/min. A 50-ml linear gradient to 100% buffer B (1 M NaCl in buffer A) was applied at 2 ml/min. The fractions containing the desired rh-GCSF were pooled and concentrated using Amicon Ultra (Millipore, USA) with a molecular mass cut-off of 5 kDa by centrifugation at 4,500 ×g at 4 ^o^C for 60 min.

Concentrated protein solution was applied to a Superdex 75 (16/60) column (GE Healthcare, USA), which had equilibrated with GFC buffer (20 mM sodium acetate, pH 5.0). Isocratic elution was performed with buffer A at 0.8 ml/min, and 2.4-ml fractions were collected. Fractions containing rh-GCSF were pooled and stored at -80°C, and protein concentration was determined based on the calculated extinction coefficient of 15820 M^-1^cm^-1^ at 280 nm for rh-GCSF. 

***Circular dichroism measurement. ***The purified rh-GCSF alongside innovator product (Neupogen®) was analyzed with 10-µM protein in 10 mM citrate buffer (20.5 ml citric acid and 29.5 ml sodium citrate, pH 5) at 22ºC by Jasco J-715 spectropolarimeter using 2 mm path length cylindrical cell [27]. 

***Analysis of monomer and aggregates. ***Size exclusion chromatography was carried out by using TSK-GEL G3000SWXL (300 mm × 7.8 mm, Tosoh, Japan) column chromatography system with photodiode array detector. The mobile phase consisted of K_2_HPO_4_-Na_2_HPO_4_ 1 mM in water with pH of 6.2. Flow rate was maintained at 0.6 ml/min, and analysis was carried out at the wavelength of 280 nm [28].

***Bioactivity assay. ***In this study, *in vitro* biological activity assay of rh-GCSF was evaluated by proliferation of HL-60 treated with DMSO. At first, 100 μl of HL-60 cell suspension (2 × 10^5^ cell/ml) was added into a 96-well microplate. Then DMSO and RA 

**Fig. 1:**
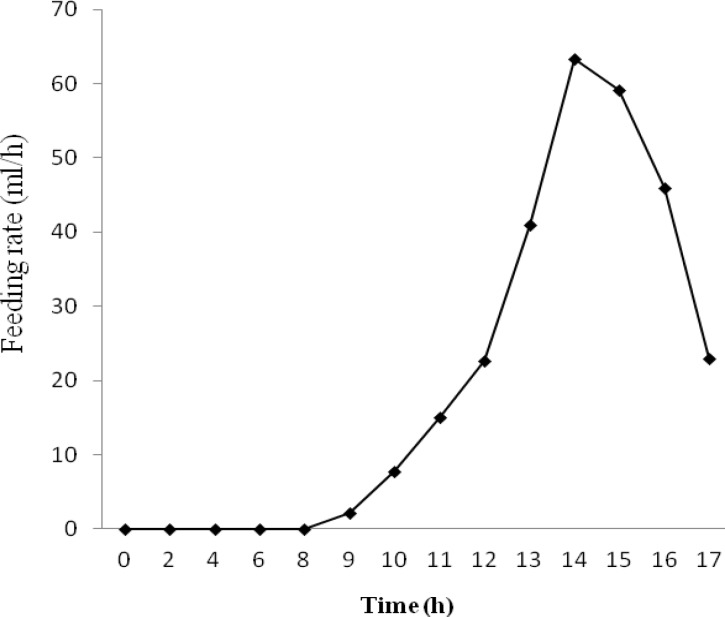
Feeding rate variation during fed-batch cultivation of *E. coli* BL21(DE3) [pET23a-*gcsf*].

(1.3% and 0.1 µM, respectively) were added to the each well and incubated at 37^o^C for 2 days in 5% CO_2_. Different dilutions of rh-GCSF were added to each well and incubated for two days at the same conditions. The reversal rate of the cells from the differentiative to the proliferative phase was determined by MTT assay. Finally, cell proliferation was measured by reductase mitochondrial enzyme through the reduction of MTT to Formazan [3].

## RESULTS

Due to metabolic burden of recombinant protein overexpression on *E. coli* as well as decrease in specific growth rate during induction [29], experiments were designed to obtain a feeding rate, which would lead to the higher attainable specific growth rate and consequently, higher cell concentration before induction. In this study, feeding rate was increased stepwise by maintaining the glucose concentration within a permissible range and using the maximum oxygen transfer capacity of the bioreactor. Figure 1 shows feeding rate variation during fed-batch cultivation of *E. coli* BL21(DE3) [pET23a-gcsf]. Maximum oxygen transfer rate was achieved by increasing the impeller rotation speed from 400 rpm to 1,200 rpm during the process. 

The exact equation for time variation in the specific growth rate was correlated by using experimental data obtained from various fed-batch cultures of *E. coli * BL21 (DE3) [pET23a-*hgcsf*] under the glucose non-limited conditions (equation 2):

(2) $$µ\left(t\right)=-0.967{\left(t-t_{0}\right)}^{3}+36.21{\left(t-t_{0}\right)}^{2}-432.2\left(t-t_{0}\right)+167.2$$

**Fig. 2:**
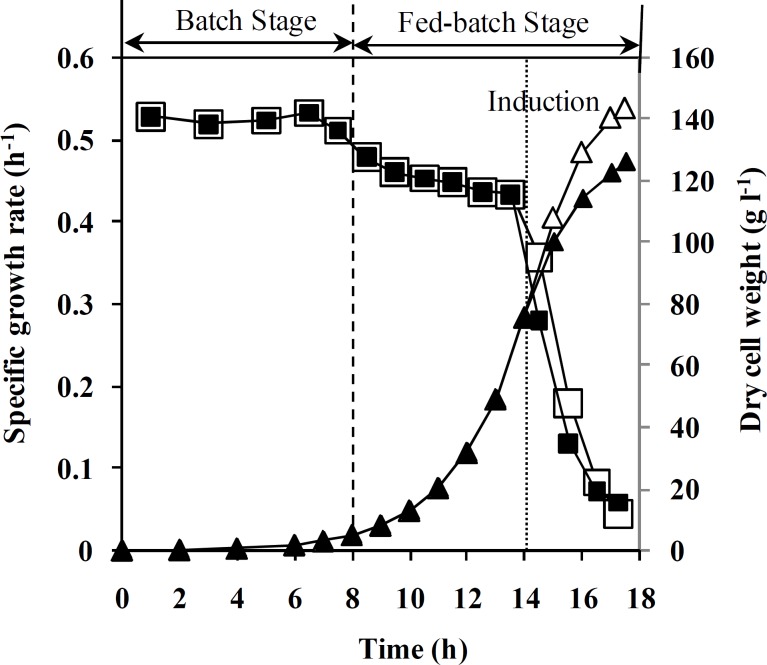
Effect of feeding strategy on dry cell weight and specific growth rate during fed batch cultivation. Specific growth rate (h^-1^) in un-induced (□) and induced processes ( ) as well as dry cell weight (g.l^-1^) in un-induced (Δ) and induced processes (▲).

$R^{2}=0.97,\mathrm{0.04}<µ<0.54$

t_0_ is time at the start of feeding, t is the time of process, and R^2^ is coefficient of determination for equation 2. Equations 1 and 2 were used to determine the feeding rate in the fed-batch process. Figure 2 shows the results of using this feeding strategy on fed-batch cultures of un-induced recombinant *E. coli *BL21 (DE3) [pET23a-*hgcsf*].

**Fig. 3:**
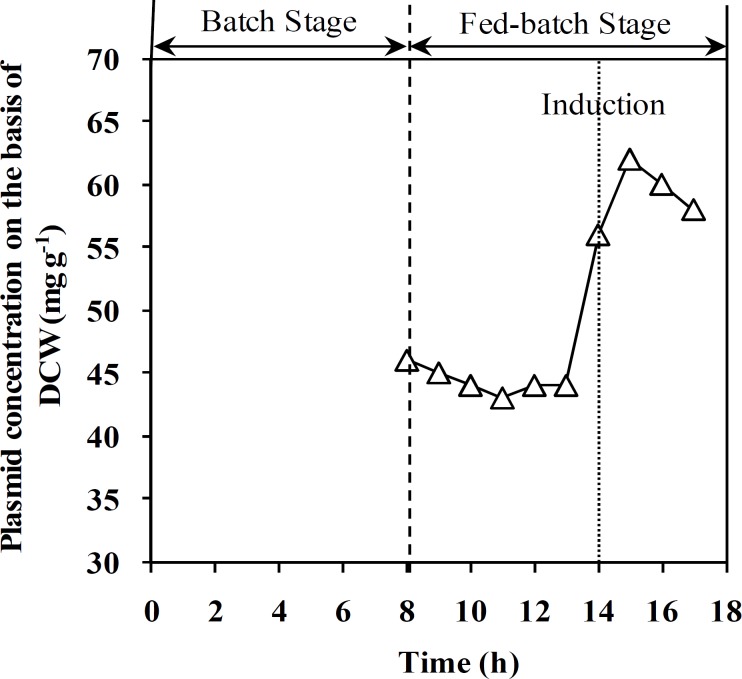
Effect of feeding strategy on plasmid concentration on the basis of CDW during fed-batch process

**Fig. 4:**
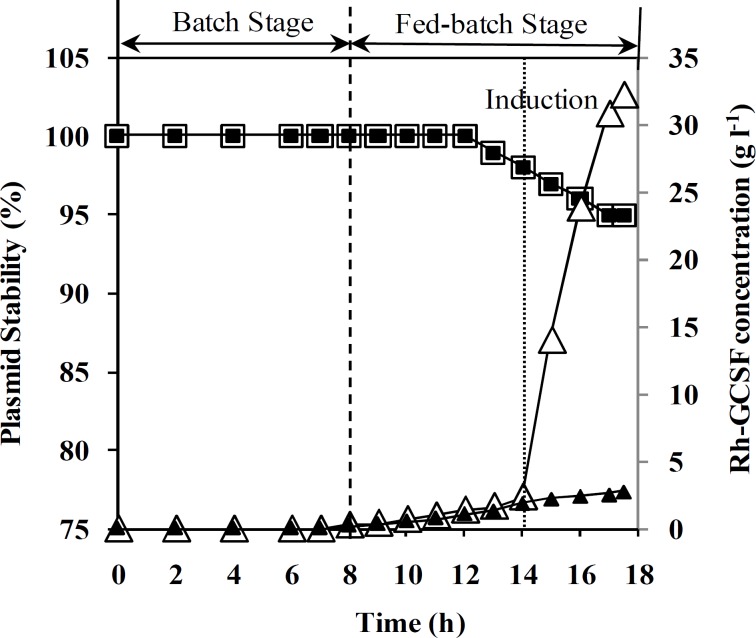
Effect of feeding strategy on plasmid stability and rh-GCSF concentration during fed batch cultivation. Plasmid stability (%) in un-induced (□) and induced processes ( ) as well as rh-GCSF concentration (g.l^-1^) in induced (Δ) and un-induced processes (▲).

Figure 2 shows DCW and specific growth rate change during both un-induced and induced fed-batch culture of recombinant *E. coli.* Final cell concentration of un-induced fed-batch culture after 17.5 ± 0.5 was 145 ± 5 g (DCW) l^-1^. Based on the results, cell density in both fed-batch culture reached 75 g (DCW)l^-1^ during the first 14 h, while specific growth rate was decreased from 0.55 to 0.43 h^-1^. During feeding stage of fed-batch cultivation, the specific growth rate was decreased from 0.55 to 0.04 h^-1^. Moreover, decrease in specific growth rate of induced fed-batch culture was more than un-induced one. Maximum cell density and rh-GCSF concentration in induced process were 125 ± 5 g(DCW) l^-1^ and 32 ± 1 g(GCSF) l^-1^, respectively. In addition, the final specific yield (Y_P/X_) and productivity of rh-GCSF in induced fed-batch culture were 250 ± 10 mg(GCSF) g^-1^ (DCW) and 1.83 ± 0.05 g (GCSF) l^-1^ h^-1^, respectively. Plasmid concentration in pre-induction stage of fed-batch culture was maintained constant and increased after induction (Fig. 3). As illustrated in Figure 4, plasmid stability was higher than 95% entire process. A slight decrease in plasmid stability after induction was related to metabolic burden of recombinant protein over-expression on host cells. 

Rh-GCSF concentration is also illustrated in Figure 4.

**Fig. 5:**
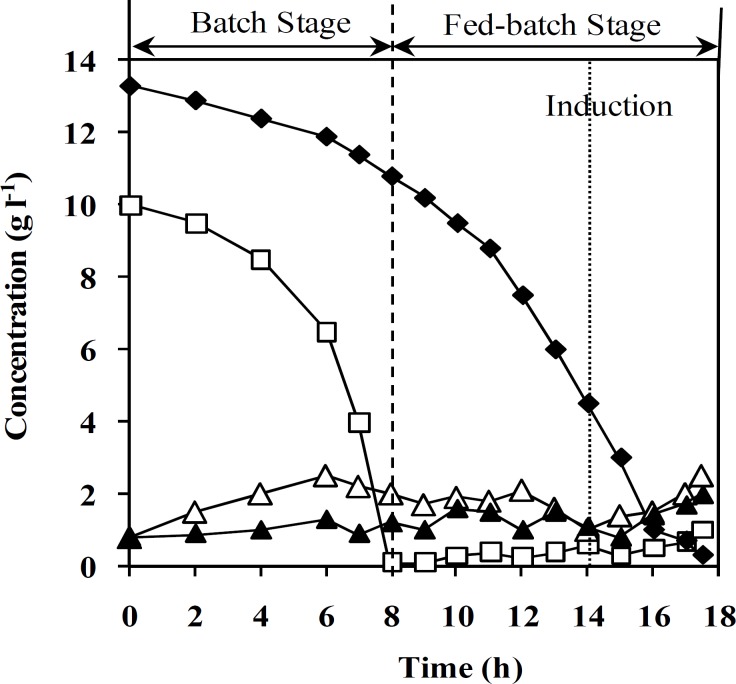
Effect of feeding strategy on concentration of the main medium components (g l^-1^). Concentration of phosphate ( ), glucose (□), acetate (Δ), and ammonium (▲), in induced recombinant *E. coli* BL21 (DE3) [pET23a-*hgcsf*].

Acetate, glucose, ammonium, and phosphate concentration were monitored during fed-batch cultivation of recombinant *E. coli *(Fig. 5). The concentration of all mentioned components was less than the inhibitory level. It is obvious that specific growth rate change before induction is negligible, while it declines sharply after induction. 

**Fig. 6:**
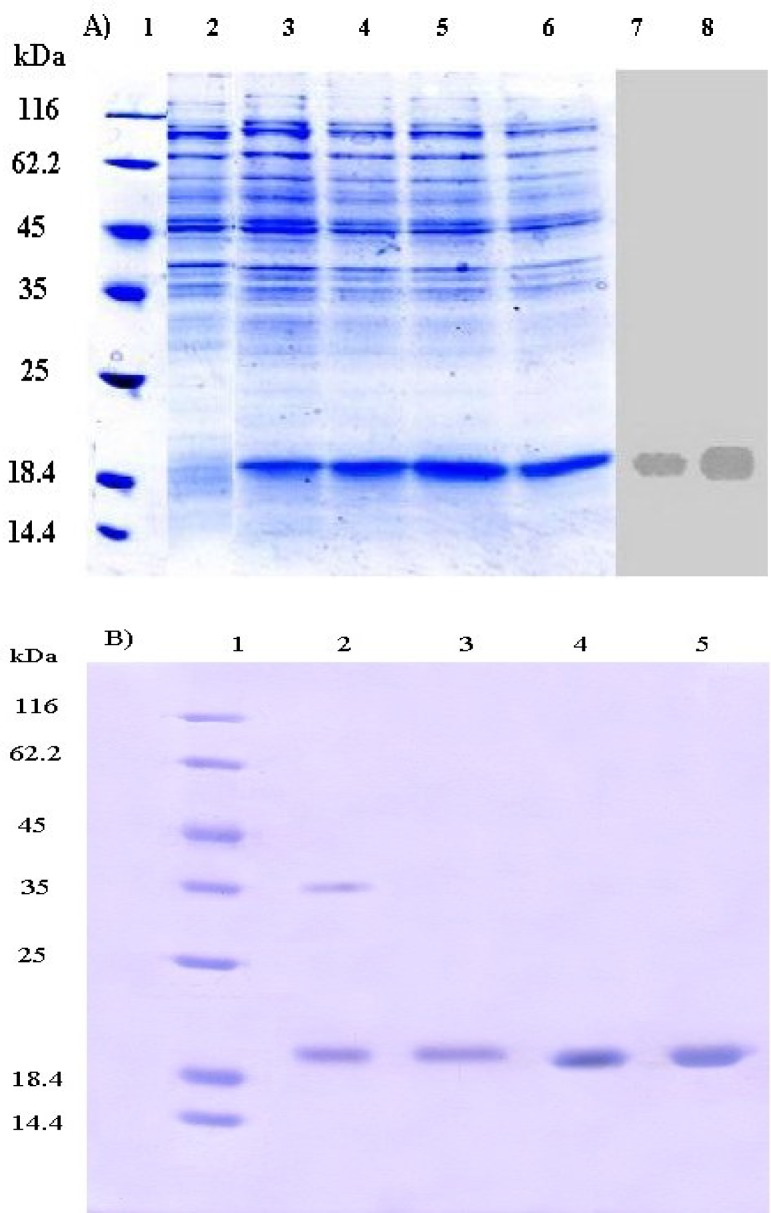
SDS-PAGE analysis and Western blott of equivalent gel of the total cell lysate of *E. coli* BL21 (DE3) [pET23a-*hgcsf*]. (A) The end of the optimized fermentation. Lanes 1-6, Coomassie-stained SDS-PAGE; Laned 7 and 8, Western blott of equivalent gel using antibody to detect the rh-GCSF; lanes 1, molecular weight markers (kDa); lane 2, total cell lysate at the induction time (t = 0 h); lane 3, total cell lysate at t = 1 h; lane 4, total cell lysate at t = 2 h; lane 5, total cell lysate at t = 3 h; lane 6, total cell lysate at t = 3.5 h. 1: The purity profile of rh-GCSF expressed in *E. coli*. (B) SDS-PAGE (15%) analysis of purified rh-GCSF showing a single protein band. Lane 1, Molecular weight marker (#SM0431, Fermentas), lane 2, refolded rh-GCSF; lane 3, purified rh-GCSF; lane 4, reference standard (Neupogen®); lane 5, reference Standard (PD-Grastim).

The expression of rh-GCSF before and after induction in fed-batch culture of *E. coli* was confirmed by SDS-PAGE and blotted GCSF onto PVDF membrane (Fig. 6). Operating on maximum achievable specific growth rate and suitable fermentation condition resulted in an appropriate expression level of GCSF. 

Purity of final product was more than 99% (Fig. 6B), which was validated by comparison with Neupogen® (Roche, Germany) and PD-Grastim (Pooyesh Darou, Iran) as reference standards. In addition, two-step washing procedure was sufficient to eliminate proteins and DNA of host cell (data not shown). Obtaining inspection chromatogram of TSK-GEL column for rh-GCSF and Neupogen confirmed molecular weight of rh-GCSF. 

**Fig. 7:**
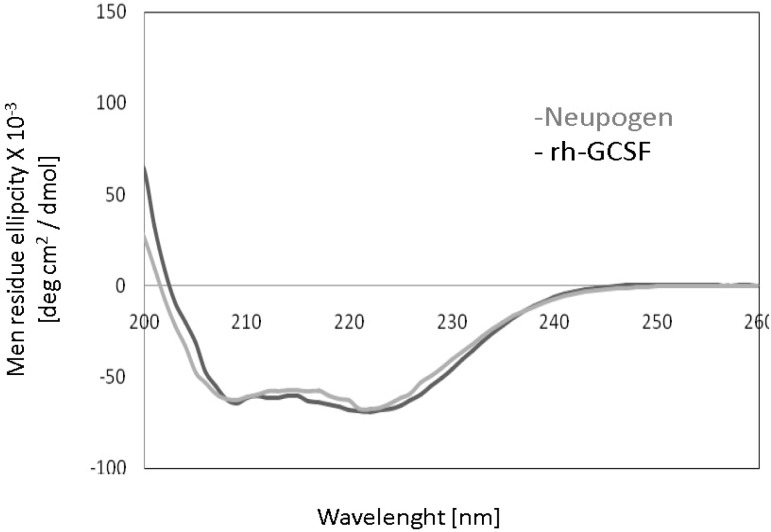
Circular dichroism spectra of rh-GCSF, and Neupogen. Circular dichroism spectra of rh-GCSF (black) and Neupogen (gray) are shown. The mean residue ellipticity of the proteins (10 mM) were measured at 22°C in 10 mM citrate buffer at pH 5.

**Fig. 8:**
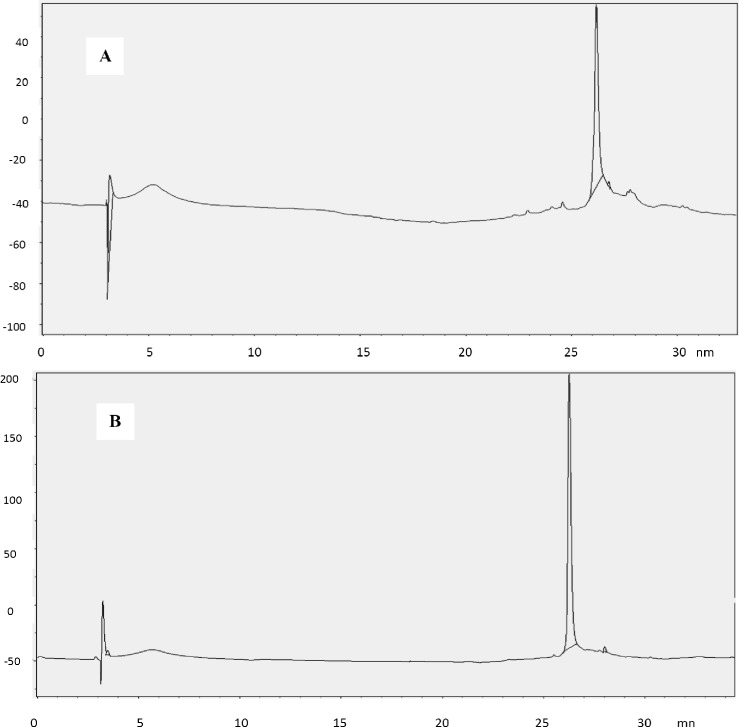
Size exclusion chromatography (SEC) spectra of rh-GCSF, and Neupogen. SEC spectra of rh-GCSF (A) and Neupogen (B) are shown. The SEC has done with TSK-GEL G3000SW_XL_ (300 mm × 7.8 mm) column. Moving phase contain 1mM K_2_HPO_4_- Na_2_HPO_4_ in water at pH 6.2.

The Bradford's assay [24] and gel densitometry analysis (Fig. 6B) showed that protein purification yield was 400 mg protein per 1 g inclusion body (40%). Based on the above results, it can be found out that the purified protein (purity > 99%) in this article is comparable with reference standards of Neupogen® (Roche, Germany) and PD-Grastim (Pooyesh Darou, Iran). In comparison with other studies, the obtained amount of recombinant protein in this article is one of the highest productivity that has been recently reported [28, 30-32]. Rh-GCSF expression and purification yield were presented in the Table 1. The circular dichroism spectra showed that the rh-GCSF was on par with the reference standard of Neupogen® (Fig. 7). The circular dichroism spectrum of rh-GCSF in sodium citrate buffer (pH 5) was consistent with the previously reported structure (PDB ID code 1RHG), indicating an alpha-helix content of 66-68% [3]. The circular dichroism spectrum of Neupogen® showed 67-70% alpha-helix content. This result indicates that the secondary structure of purified rh-GCSF is similar spectra of purified rh-GCSF and PD-Grastim (Fig. 8) showed that both proteins have a similar curve; to Neupogen®. The size exclusion chromatography therefore, it is obvious that protein purification successfully performed and purified protein has had no dimer contamination. The increase in the growth of HL-60 cells by rh-GCSF was similar to Neupogen®. Therefore, it can be concluded that the specific activity of rh-GCSF is identical with the innovator product. 

## DISCUSSION

According to the data presented in Figure 2, specific growth rates ranging from 0.55-0.4 h^-1^ were applied before induction of rh-GCSF. Implementation of the fed-batch process in higher specific growth rate before induction led to higher biomass production and consequently, higher productivity of GCSF, which was in agreement with the data presented by other researchers [14, 16, 28, 33].

Figure 2 illustrates that during feeding, the specific growth rate was decreased, which may be because of (1) inhibitory effect of acetate accumulation, (2) oxygen transfer limitation due to high cell density and accumulation of antifoam at the end of the fermentation process [12, 34], (3) stress response related to induction of recosmbinant protein overexpression in *E. coli *and environmental stresses such as very high mixing rate [8]. This Figure shows a sharp decrease in specific growth rate after induction because of the increasing metabolic burden of cells by recombinant gene overexpression [12, 34]

The results of plasmid content (Fig. 3) showed that in the employed fed-batch technique, high cell density has no negative effect on plasmid content. Furthermore, Plasmid stability is one of the most important issues affecting the productivity of recombinant protein production in *E. coli *fed-batch cultivation [31, 32]. Although it has been difficult to find real trends in relationship between plasmid stability and specific growth rate in most cases, change in this parameter seems to be a function of the specific growth rate in the fed-batch. It has found that plasmid stability is reduced by decreasing growth rate, primarily because growth rate of plasmid-containing cells is less than the plasmid-free cells. [13, 31, 32]. Therefore, it can be expected that plasmid stability increases by increasing the specific growth rate (Fig. 4).

**Table 1:** Protein expression and purification yield

	**Dry cell weight **	**Rh-GCSF concentration **	**Rh-GCSF yield **
None-induced fed-batch	142 (g/lit)	---	---
Induced fed-batch	127 (g/lit)	32 (g/l)	1.83 (mg.l^-1^.h^-1^)
Purification	---	400 (mg/g IB)	40%

IB, inclusion body

At high cell density, acetate formation and accumulation are main challenges during recombinant protein production [10, 28] that can be minimized by controlling the specific growth rate below a certain value (depending on strain) [12]. In this study, by exponential feeding strategy, specific growth rate was controlled below critical value; therefore, acetate concentration was controlled under inhibitory level (less than 5 g l^-1^ for acetate) [35] (Fig. 5). By this feeding strategy, glucose concentration was also maintained simultaneously at a suitable concentration range without any starvation and accumulation of glucose (Fig. 5). Dissolved oxygen concentration was maintained higher than least quantity that was reported throughout fed-batch mode (more than 6%, v/v air saturation) [36].

In this study, human recombinant protein expression was carried out in developed fed-batch culture of *E. coli* (data not shown). Therefore, in comparison with other studies, the cultivation time was decreased, while cell density and rh-GCSF concentration were increased significantly [6, 13, 25, 30, 31]. Overall, the product-ivity of GCSF was higher than that reported by other researchers [34].

Figure 6A shows the SDS-PAGE of total cell proteins and Western blot of equivalent gel after induction at a cell density of 75 g l^-1^ DCW with 2 mM IPTG as inducer. Fraction of rh-GCSF remarkably increases by passing time after induction, and percentage of expressed recombinant protein to total protein at the end of the process is 45.

This process has the following advantages resulting in the higher percentage of recombinant protein expression to total protein: (1) reduction of process time, (2) decrease of by-products accumulation, especially acetate, (3) increase of plasmid stability, (4) suitable concentration range of nutrients, such as glucose, ammonium and phosphate during fed-batch cultivation [7, 13, 14, 26, 37].

Applying modified exponential feeding strategy for fed-batch culture of recombinant *E. coli* resulted in an increase in attainable specific growth rate, reduce of process time, and maximum productivity. Therefore, this strategy can successfully be applied to enhance the production of any recombinant proteins in *E. coli *or other expression systems.

Based on the above results, it can be found that rh-GCSF protein isolated in this study is highly pure and comparable with the innovator products, Neupogen® and PD-Grastim. These properties serve as a basis for comparison of process reproducibility, creating the range of conditions to stabilize the protein during production and storage as well as for identifying characteristics valuable for monitoring stability during long-term storage.

In the present study, we developed an efficient procedure for production and purification of rh-GCSF in *E. coli *by using a new developed method. According to the available data, result of this article is one of the highest Y_P/X _and productivity that has been reported for any human recombinant protein expressed in *E. coli* [27, 28, 30, 34, 38].
